# Image Compression Based on Hybrid Domain Attention and Postprocessing Enhancement

**DOI:** 10.1155/2022/4926124

**Published:** 2022-03-17

**Authors:** Yuting Bao, Yuwen Tao, Pengjiang Qian

**Affiliations:** School of Artificial Intelligence and Computer Science, Jiangnan University, Wuxi 214122, China

## Abstract

Deep learning-based image compression methods have made significant achievements recently, of which the two key components are the entropy model for latent representations and the encoder-decoder network. Both the inaccurate estimation of the entropy estimation model and the existence of information redundancy in latent representations lead to a reduction in the compression efficiency. To address these issues, the study suggests an image compression method based on a hybrid domain attention mechanism and postprocessing improvement. This study embeds hybrid domain attention modules as nonlinear transformers in both the main encoder-decoder network and the hyperprior network, aiming at constructing more compact latent features and hyperpriors and then model the latent features as parametric Gaussian-scale mixture models to obtain more precise entropy estimation. In addition, we propose a solution to the errors introduced by quantization in image compression by adding an inverse quantization module. On the decoding side, we also provide a postprocessing enhancement module to further increase image compression performance. The experimental results show that the peak signal-to-noise rate (PSNR) and multiscale structural similarity (MS-SSIM) of the proposed method are higher than those of traditional compression methods and advanced neural network-based methods.

## 1. Introduction

In the age of information technology, pictures have become important information carriers, and massive amounts of image data can lead to enormous transmission and storage pressures. For example, an original RGB image with a resolution of 512 × 768 has a theoretical storage size of about 1.125 MB, and after compression, the storage size of the image is only one-sixtieth of the original image or even smaller. Therefore, image compression is crucial in computer vision, and the trend of information technology has put forward higher demands on image compression efficiency. Traditional image compression methods [1–3] have achieved better performance through finely designed manual features and complex processing. For example, JPEG [1] employs the discrete cosine transform (DCT) [4] to eliminate redundancy among pixels. Traditional compression algorithms, on the other hand, lack learning capabilities. Thanks to the advancement of deep learning, this also gives new ideas for image compression methods.

In contrast to the traditional methods, where a linear transform module is replaced by a nonlinear neural network, the performance of image compression methods is determined by how the network structure is constructed to produce more compact latent features. In addition, accurate entropy estimation is one of the pivotal factors to improve the performance of image compression. A good entropy model can better suit the true distribution of an image. A further important task is to design the quantization module, which has a significant impact on compression performance.

A classical deep learning-based image compression structure converts images into compressible latent representations by stacking convolutional neural networks [5]. These latent representations are then entropy coded through statistical redundancy, and lossless compression is performed through entropy coding to create bitstreams. At the same time, the joint optimization decoder decodes the latent representations into images. Classical learning-based image compression algorithms [6, 7] use a variational autoencoder structure, and significant progress has been made in various technical components of this architecture, including the use of generalized divisive normalization (GDN) modules in nonlinear transformers, which has been validated for probabilistic modeling and image compression tasks. A nonlocal model was used in the literature [8], and a CNN-based wavelet transform was used in the literature [9] to reduce redundancy among pixels. The entropy estimation model with hyperprior was first proposed in the literature [7] to capture the hidden information of latent representations, aiding the generation of entropy model parameters and improving the mismatch between entropy model and hidden marginal distributions. In the literature [10, 11], the autoregressive module was introduced, and the autoregressive and hyperprior modules complement each other to improve the entropy estimation. The literature [12] proposed a parallelizable checkerboard grid context model and changed the decoding order so as to speed up decoding without breaking performance. The literature [13] used importance maps to guide image compression based on generative adversarial network loss functions for enhancing the subjective image quality. The literature [14] used a manually set distortion weight map to control the bit-rate allocation and assigned a larger distortion weight to the region of interest in the image, thus enhancing the quality of the region of interest. The problem that DNN-based networks cannot be directly used for near-lossless coding is addressed in the literature [15]. It is possible to see that the design and improvement of the deep neural network-based image compression methods focus on improving the main encoder-decoder, the quantization module, and the entropy estimation module structures.

In addition, improving the image compression quality is the eternal theme of image coding and decoding. We need to understand that enhancing the image quality at the decoding end is equivalent to improving the compression efficiency. Moreover, since the actual lossy compression standards are not theoretically optimal, there are information redundancies that can continue to be explored and utilized. Furthermore, we find that there are still some limitations in the existing methods. For example, the information transmitted using a hyperprior is not standardized and fully utilized. This part of the information, which is encoded into the bitstream to construct the entropy model, is not used for image reconstruction. We want to eliminate compression artifacts and blur, despite the fact that existing compression algorithms offer decent compression efficiency. The purpose of this study is to investigate how to build an effective learning-based image compression approach. To summarize, the following are the study's innovations:

- This study describes a hybrid domain attention mechanism that was embedded into the transform encoder-decoder and hyperprior module to output latent representations and hyperpriors with channel global context-adaptive activation. Our hybrid domain attention mechanism was embedded into different network layers, which is not only applied to quantized latent representations. The created attention mask dynamically analyzes the relevance of the features to be compressed through the deep learning-based architecture and allocates more bits to more essential features, which will help increase the entropy coding efficiency.
- In addition, because quantization poses a zero-gradient problem, the training of a deep learning-based network cannot be carried out. We use the hybrid quantization approach to fix this problem, that is, the forward propagation adopts the rounding function and the backward propagation adopts the form of straight propagation. Apart from the nondifferentiable problem, quantification also leads to a loss of information. This study proposed an inverse quantization module to alleviate the errors induced by quantization.
- Finally, as our image compression method is lossy, the reconstructed images will inevitably contain compression artifacts. In order to further increase image compression quality, obtain results with rich texture information, and generate vivid details, we perform a filtering process on the compressed image.

## 2. Related Work

### 2.1. Traditional Image Methods

Traditional image compression standards use artificially designed encoders, such as the discrete cosine transform used by JPEG [1], separating high-frequency information from low-frequency information and allocating bits according to signal importance to reduce information redundancy. To improve compression performance, entropy coding is also used. The coding of a source according to its probabilities is known as entropy coding. The process of entropy coding is lossless, i.e., there is no loss of image information. Entropy coding includes Huffman coding, tour coding, and arithmetic coding.

### 2.2. Deep Learning-Based Methods

Deep learning-based image compression is not independent of traditional image compression and is more built on top of it. There are two main approaches, a convolutional neural network-based compression approach using automatic encoders and a compression method with postprocessing filtering combined with traditional encoders. Traditional image compression uses transform coding that introduces block effects and compression artifacts, and while many methods can deal with these issues well, deep learning has a superior ability to solve these types of problems. For the design of transform encoder-decoder, some works [16, 17] used recurrent neural networks (RNNs) to achieve recursive compression of residual information, while other parts of works [6, 7, 10, 11] used stacked convolutional blocks to achieve this. Considering the limited perceptual field of convolutional blocks, Cheng et al. [18] used residual blocks to increase the perceptual field. Rippel and Bourdev [19] proposed the use of feature pyramid pooling (FPN) to obtain more powerful feature representations. In addition, since convolutional operations share features, this would lead to information redundancy. Li et al. [20] proposed using an importance map to adjust the bit allocation of images. The importance map is derived by training a branch of a 3-layer convolutional network. However, according to the method in [20], the explicit learning content needs weight, which increases the computational overhead, and it is difficult to adaptively allocate bits for deep features. Attention mechanisms have demonstrated considerable strength in adaptive learning of feature importance in recent years. In tasks such as natural language processing [21] and semantic segmentation [22], significant results have been made. Furthermore, introducing nonlocal block (NLB) into neural networks can greatly increase image denoising and image super-resolution reconstruction performance [23, 24]. This study, therefore, presents a hybrid domain attention mechanism that is embedded into both main and hyper coders, which allows features to have adaptive responses, reinforcing important features, weakening unimportant ones, and further improving the compression performance. Akbar et al. [25] employed multiplicative convolution. The use of multiplicative convolution is also based on the idea of allocating more bits to important regions and reducing spatial redundancy.

Because deep learning-based methods require network the training, quantization operations are not differentiable. The literature [6] proposed the use of adding uniform noise as an alternative to true quantization. The literature [11] sets the gradient of the quantization operation to a fixed value to ensure that deep learning training takes place. To make the quantization smoother, a soft-to-hard quantization is utilized instead of direct scalar quantization in the literature [26].

In the entropy coding section, different entropy models are proposed for the quantized latent representations. In the earlier literature [6], an entropy model constructed by a linear segmentation function was used for bit-rate estimation, the model has fixed parameters of its probabilistic model at the end of training, and the quantized latent representations are entropically encoded and entropically decoded based on these probabilities. To improve the entropy model, a hyperprior structure was proposed in the literature [7], in which the authors used a Gaussian-scale mixture (GSM) (Gaussian with different means and scales) modeling approach to estimate the probability of latent features. While the parameters needed for GSM modeling are derived by the hyperprior module, the latent features are then encoded into the bitstream and sent to the decoder. This achieves image adaptive coding and also obtains better performance than BPG (4 : 4 : 4) [3]. In the literature [10], an autoregressive context module is proposed to perform parameter prediction of the entropy model in conjunction with the hyperprior structure proposed in the literature [7].

To enhance the image reconstruction quality even more, many research studies [26, 27] used generative adversarial network (GAN) as a distortion measurement in the training phase to guide the decoder to generate more realistic texture structures, resulting in reconstructed images with good subjective quality, but the texture structures generated in this way are not real textures and do not have fidelity. For this reason, TuCode technology in [28] proposed an enhancement module that acts on full-resolution images to reduce compression artifacts in images by building a simple neural network to filter the reconstructed images. The literature [29] investigated the effect of decoding network complexity on image compression performance and concluded that postprocessing networks do not significantly improve compression performance when the network at the decoding end has a strong enough reconstruction capability. When using neural networks for traditional encoding schemes, the auxiliary information generated during the encoding process has a significant effect on image denoising, and ByteDance uses predictive information and coding unit (CU) block segmentation information to assist the neural network in replacing the deblocking (DB) and sample adaptive offset (SAO) modules for loop processing. Qualcomm uses a network of BS-assisted information to replace the DB filter for block filtering.

## 3. The Proposed Image Compression Method

### 3.1. Image Compression Architecture

**Figure 1:**
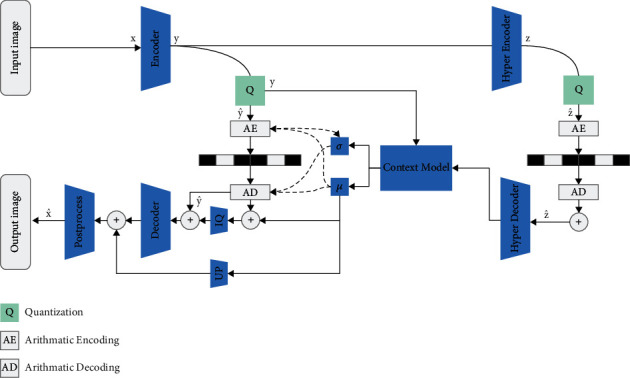
Network frame architecture of the proposed method.

Figure 1 shows the network structure. We deploy a deep learning-based automated encoder network. This method mainly includes the main encoder-decoder, hyper encoder-decoder, autoregressive context module, and postprocessing enhancement module. In particular, given the training images **x**, a transformer encoder generates corresponding latent features **y**. The quantization quantizes the latent features to $\hat{y}$, and subsequently, entropy codes the quantized $\hat{y}$ into a bitstream for transmission. The autoregressive context module combined with the hyperprior module is used for entropy coding. The entropy coding in this way will first estimate the distribution of latent representations **y** through the hyperprior network, and the output of the hyperprior encoder will be then quantized and encoded into bitstream. The reason why it will be encoded into the bitstream is that this part of the bitstream is required during decoding, and the accurate entropy model will improve the compression efficiency. The use of autoregressive prior information to estimate the distribution of latent representations **y** is to capture an accurate entropy model.

The attention module is used to adjust bit allocation based on the importance of the information. Considering that the goal of the image compression algorithms is to obtain the highest possible quality reconstructed image for a given bit-rate target, the method in this study adds a postprocessing enhancement module, and the image decoded by the main decoder and the mean information generated for modeling the Gaussian distribution are jointly fed into the postprocessing network to assist in generating the final reconstructed image.

We can deduce from our understanding of information theory that when the required coding features are concentrated, the smaller the value of information entropy, the fewer bits are required, and the resulting reconstructed image has more distortion. On the other hand, when the information entropy of the features is higher, the more bits are required, and the image distortion is lower; therefore, we need to use the hyperparameter *λ* to establish a trade-off between the two. The entire training of the compression method is optimized by means of the following loss function [30]:

(1) $$\begin{array}{c} \text{Loss}=\mathrm{\lambda D}\left(x,\hat{x}\right)+R_{\hat{z}}+R_{\hat{y}}, \end{array}$$

where *R* refers to the bit rate, and *D* indicates the distortion between the images before and after compression. There are two commonly used distortion metrics, namely, multiscale structural similarity (MS-SSIM) and mean square error (MSE) [31]. The distortion and bit rate are weighed by. *λ*.

In training, we use an entropy estimation approach consistent with the model in the literature [10], and we model the latent features as follows:

(2) $$\begin{array}{c} p_{\hat{y}|\hat{z}}\left(\hat{y}|\hat{z}\right)=\prod_{i}Ν\left(\mu^{i},\sigma^{2\left(i\right)}\right)*u\left(-\frac{1}{2},\frac{1}{2}\right)\left({\hat{y}}_{i}\right). \end{array}$$

Each latent representation ${\hat{y}}_{i}$ is modeled as a Gaussian distribution with *μ*^*i*^ and *σ*^*i*^, which are forecasted by the distribution of the hidden variable $\hat{z}$. $\hat{z}$ is called the hyperprior, *u*(·) denotes a uniform distribution, and *∗* denotes the convolution operation. Because prior information about $\hat{z}$ does not exist, we model the hyperprior $\hat{z}$ as follows:

(3) $$\begin{array}{c} p_{\hat{z}|\psi }\left(\hat{z}|\psi \right)=\prod_{i}\left(p_{z^{i}|\psi^{\left(i\right)}}\left(\psi^{\left(i\right)}\right)*u\left(-\frac{1}{2},\frac{1}{2}\right)\right)\left({\hat{z}}_{i}\right), \end{array}$$

where *p*_**z**^(*i*)^*|ψ*^(*i*)^_ represents each univariate's distribution, and *ψ*^(*i*)^ represents the parameters of this distribution. Finally, the bit rate in our method consists of the bit rate of the latent representations $\hat{y}$ and the bit rate of the hidden variable $\hat{z}$. These bit rates are denoted as follows:

(4) $$\begin{array}{c} R_{\hat{y}}=\sum_{i}-\;\log_{2}\left(p_{{\hat{y}}_{i}|{\hat{z}}_{i}}\left({\hat{y}}_{i}|{\hat{z}}_{i}\right)\right),\\ R_{\hat{z}}=\sum_{i}-\;\log_{2}\left(p_{{\hat{z}}_{i}|\psi^{\left(i\right)}}\left({\hat{z}}_{i}|\psi^{\left(i\right)}\right)\right). \end{array}$$

Table 1 shows the network architecture and related parameters for separate components in our compression method. One of them is the hybrid domain attention mechanism (HDAM), which will be described in Section 3.2. In addition, the inverse quantization module will be described in Section 3.3 and the postprocessing module will be described in Section 3.4.

### 3.2. Hybrid Domain Attention Mechanism

**Figure 2:**
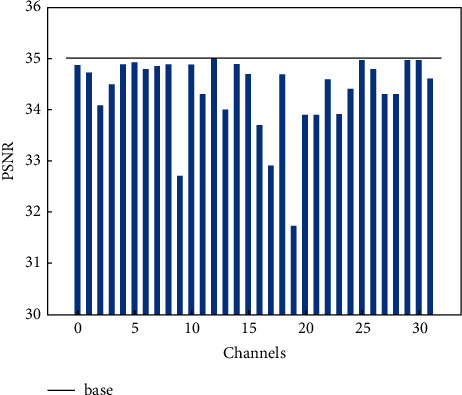
Channel influence on reconstruction distortion (PSNR degradation of the first 32 channels).

**Figure 3:**
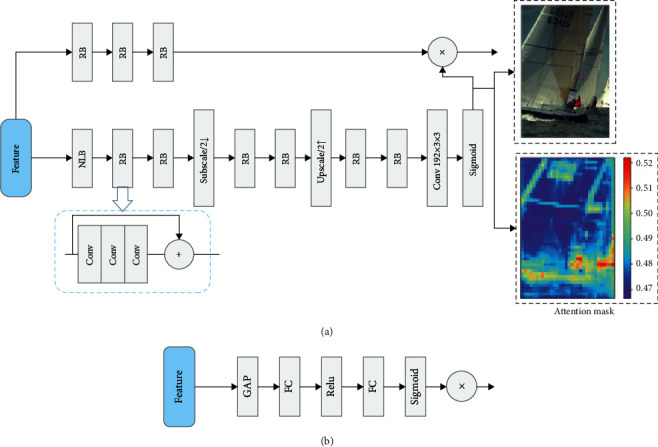
(a) The structure of spatial attention in hybrid domain attention module. “RB” is for resblock. “NLB” is for nonlocal module proposed in [34]. (b) The structure of channel attention in hybrid domain attention module. “FC” is for 2-layer fully connected layer. “GAP” is for global average pooling.

In previous studies, transform encoders were often implemented using stacks of convolutional neural networks. In learning-based image compression, learning a transform encoder with less redundant information and more critical reconstruction information through convolutional neural networks is one of the keys to better compression performance. While the convolutional layers are limited by the range of perceptual fields and have only local bias induction capabilities, increasing the depth of the network allows for deeper dependencies, but at the same time brings with it a significant increase in computational effort. Many recent studies have used attention mechanism modules to improve image recovery and compression performance [8, 32], where attention modules were introduced into transformers to model the global dependencies between features, resulting in a less redundant latent representation of the image on the transformer encoder side. The attention mechanism starts from the human visual mechanism by adaptively learning the weights of different features and acquiring the areas that need to be focused on. Previous works have used only spatial information to generate attention maps, which do not allow for good mining of correlations between channels of latent features. As shown in Figure 2, we experimented with the effect of the first 32 channels of the latent representations on the distortion performance of the reconstructed images and came to the conclusion that each channel has a different importance in the final reconstruction effect. Unlike previous works and inspired by [33], we proposed a hybrid domain attention mechanism that works in both the channel and spatial domains. The hybrid attention module is embedded in the transformer encoders and adaptively learns relevant compression features to obtain a transformer encoder with reduced information redundancy and with reconstructed key information. In our hybrid domain attention mechanism module, the input features are first passed through the channel attention module to obtain the channel domain attention map, which is then multiplied element by element with the input features to obtain the features required by the spatial attention mechanism. The output of the channel domain is utilized as the input feature map for the spatial attention mechanism, and then, the spatial domain attention map and the input features are multiplied element by element to obtain the final output.  Figure 3(a) depicts the architecture of the channel attention mechanism, and the channel mask can be described as follows:

(5) $$\begin{array}{c} A_{c}\left(X\right)=\sigma \left(\text{MLP}\left(\text{AvgPool}\left(X\right)\right)\right), \end{array}$$

where **X** is the input feature map, *A*_*c*_(·) denotes the channel domain attention map, MLP stands for a 2-layer neural network, and *σ* represents the sigmoid activation function. The spatial information of the aggregated feature map **X** is first derived by averaging pooling over spatial dimensions of the feature map, the spatial information is then sent to the MLP to compress its spatial dimensions, and then it undergoes a sigmoid activation operation to create the feature map of channel attention.

The spatial attention module is divided into a backbone branch, which uses traditional residual blocks to generate features, and a mask branch, in which the nonlocal block (NLB) [34] is embedded.  Figure 3(b) depicts the spatial attention mechanism (b), and the spatial mask can be described as follows:

(6) $$\begin{array}{c} A_{S}\left(X\right)=\sigma \left(F_{\text{NLB}}\left(X\right)\right), \end{array}$$

where *A*_*S*_(·) represents the attention mask, and *F*_NLB_(·) denotes the result of utilizing *NLB* and the subsequent residual blocks and convolution.

As shown in Table 1, we integrate hybrid domain mechanisms into the framework of the proposed method. The attention module aids the network's global adaptive response by reinforcing essential features while weakening unimportant ones, thus implicitly learning feature importance mapping and delivering more bits for textured regions, resulting in better visualization with the naked eye at similar bit rates.

### 3.3. Hybrid Quantization

For lossy image compression, all features need to be quantized into integer form for entropy coding. Deep learning-based training, on the other hand, is hampered by the inherently nondifferentiable nature of quantization. In the training phase, quantization methods for deep learning-based image compression methods [6, 7] typically take the form of approximate quantization by adding uniform noise for end-to-end optimization:

(7) $$\begin{array}{c} Q\left(y\right)=y+\Delta \left(-\frac{1}{2},\frac{1}{2}\right). \end{array}$$

The other part of the work uses straight-through estimation (STE) of the gradient and manually sets the backward propagation expression for the rounding function:

(8) $$\begin{array}{c} \frac{d}{d_{y}}\left\{y\right\}≔\frac{d}{d_{y}}\left[\left\{y\right\}\right]\\ =\frac{d}{d_{y}}y=1. \end{array}$$

In our method, we integrate two quantization methods. For the encoder output **y**, we use STE quantization to round up and feed the quantized one into the decoder, while for the entropy model network, we use approximate quantization with added noise for entropy modeling. In addition to this, to reduce the loss of information due to quantization, we incorporate an inverse quantization subnetwork. We treat the loss of floating-point numbers due to rounding operations as being added to the noise in the range of (−0.5, 0.5), which is why most compression methods today use an approximate quantization operation in the form of added noise. The inverse quantization network is similar to existing image denoising efforts, making the inverse quantized feature data as close as possible to the prequantization data.

**Figure 4:**
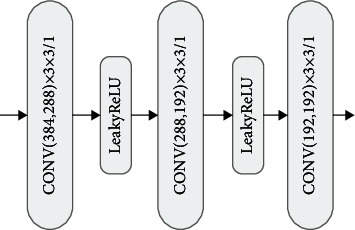
The architecture of our dequantization network.

Figure 4 depicts the specific network structure. The addition of uniform noise treats the loss of floating-point numbers due to quantization as a random form of noise, but the loss due to rounding is actually traceable, which also helps the inverse quantization network to perform a more accurate “denoising job.”

### 3.4. Postprocessing Module

Since image compression approaches based on deep learning are lossy, the model in the form of hyperprior needs to ensure that the dimensionality of the latent representation is low; otherwise, the latent representation itself may contain redundancy, which will result in inevitable compression artifacts and poor compression performance. In this case, the hyperprior module may have some loss of information that affects the accuracy of the parameters required to model the entropy rate, especially for high bit rates and high resolutions. Taking into account the degradation information in image compression and the necessity to improve the compressed image's quality and provide better visual effects, we proposed introducing the postprocessing module into the main decoding end. In addition, we proposed reuse of the mean information *μ* derived from the hyperprior module joint autoregression module. As shown in equation (2), through the training of the neural network, *μ* will capture the hidden information in $\hat{y}$, so *μ* contains rich structural information. To exploit the full potential of the auxiliary information, we feed the mean information into the postprocessing module to further assist the postprocessing network in removing compression artifacts.

**Figure 5:**
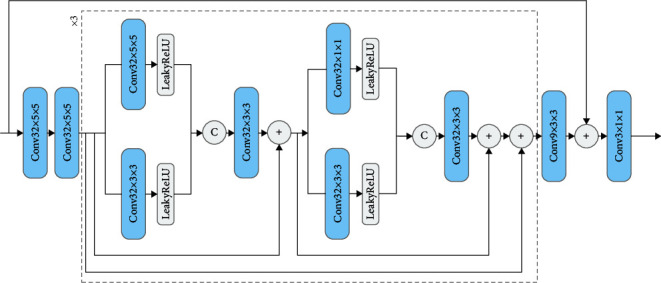
The architecture of our postprocessing module. “C” is for concatenation.

As inspired by image noise reduction and super-resolution network design strategies [35], our postprocessing network uses a residual network structure for quality enhancement of the reconstructed image. As illustrated in Figure 5, we started by adding two convolutional layers to get shallow features, and at the same time, the number of channel dimensions was changed from 6 to 32 and then cascaded through three identical modules for detailed enhancement. Because we are dealing with full-resolution images, we use only three enhancement blocks in order to keep the computational cost from rising as the network depth grows. The enhancement blocks are added with two multiscale residual blocks to extract multiscale features. In addition, three different convolutional kernel sizes are used in these blocks, including 5 × 5, 3 × 3, 1 × 1, and PReLU activation functions, and finally, the convolution is used to change the channel dimension in the image before implementing global residual learning to obtain the enhanced image.

In fact, a general postprocessing module is not necessary because it will fail if the decoder network is powerful enough [29]. However, our postprocessing can increase the quality of the decoded images even further due to the reuse of information from *μ*. In the ablation experiments in Section 4.3, we proved the effectiveness of the postprocessing module we offered.

## 4. Experiments

### 4.1. Operation Details

1. Experimental environment. We used PyTorch to implement our method, and we ran all of our tests on an NVIDIA-2080TI GPU with 11 GB of video RAM.
2. Experimental data. To ensure that the recommended technique is effective, we conducted a series of tests. Since image compression tasks are unsupervised tasks that do not require additional label files, most of the image compression methods crawl high-resolution image data on the web as the dataset for the training model. We used 20,745 high-quality images provided by Flick.com. After preprocessing, these images were randomly cropped to size 256 × 256, and a total of around 800,000 images were obtained as the training set after preprocessing. To assess the efficiency of image compression methods, we tested the performance of various image compression methods using the Kodak Photo CD image dataset [36] as testing data.
3. Comparison of methods. Our comparative experiments include classical traditional image compression methods (JPEG2000 [2], BPG [3]) and more recent deep learning-based image compression methods (Ballé et al. [6, 7], Minnen et al. [10], Lee et al. [11]). JPEG2000 [2] compression was tested using the official test model OpenJPEG 2000 configured in YUV 420 [37] and was implemented, and BPG [3] compression was tested using the BPG software [9] in the format YUV 440.
4. Our method. To plot the rate-distortion performance curve, we trained different models according to different distortion measures. In the models with PSNR as the metric, we employed MSE as the distortion function *D* in the loss function, while in the distortion metric with MS-SSIM, we used (1-MS-SSIM) as a function of D. Six models are trained for each distortion scale based on different bit rates, for a total of twelve models, where the size of the bit rate is controlled by hyperparameter *λ*, we use different values (256, 650, 2048, 4096, 6144, and 8192) to train our models, and each *λ* corresponds to a rate-distortion point. The performance of the image compression method is represented by the line obtained by connecting each of the rate-distortion performance points. Initially, we assigned *λ* to 8192 for training and the model's batch size to 16, we used the Adam optimizer [38] for parameter optimization, the starting learning rate is 1 × 10^–4^, and because the learning rate is updated using the callback function 'ReduceLROnPlateau' in the PyTorch framework, it will reduce the learning rate after the loss is no longer reduced. For the other rate-distortion points, we used the initially trained model as the pretrained model for the next training and fine-tuned the initially trained model to obtain the other rate-distortion points while keeping the training settings the same. In the model with MS-SSIM as the distortion metric, we used the original model with PSNR as the distortion metric as a pretraining model and changed the distortion metric in the loss function in this model. In addition, we set *λ* to 384 and the other rate-distortion points in the model with MS-SSIM as distortion metric on top of this pretraining model. The values of *λ* are, respectively, 16, 32, 64, 128, 256, and 384.
5. Evaluation metric. Bit per pixel (BPP) reflects an image's compression ratio. For images with the same aspect ratio, the smaller the BPP, the higher the compression ratio. For the image quality evaluation indicators, PSNR and MS-SSIM indicators are used to evaluate, respectively. PSNR is based on element-by-element comparison of differences, does not take into account human visual perception, and lets alone human esthetics, so PSNR can reflect pixel-level distortion, while MS-SSIM measures image similarity in terms of luminance, contrast, and structure, respectively.

### 4.2. Compression Performance

**Figure 6:**
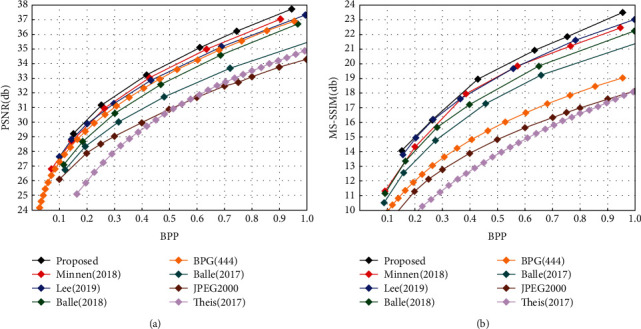
Rate-distortion efficiency. (a) Rate-distortion plots for several methods on Kodak using the PSNR metric. (b) Rate-distortion plots for several methods on Kodak using the MS-SSIM metric.

The unit of PSNR and MS-SSIM is db, and a higher value means less distortion and better visual impact. As shown in Figure 6 our rate-distortion curves plotted using PSNR (see Figure 6(a)) and MS-SSIM (see Figure 6(b)) as distortion metrics shows that our method has significant performance advantages over JPEG 2000 [2], BPG [3], Ballé2018 [7], and at the same BPP for both of the evaluation indicators mentioned. Our technique exhibits equivalent performance gains to Minnen [10] at low bit rates, and intuitionistic performance increases above Minnen [10] at large bit rates. Furthermore, our method improved performance at all bit rates, demonstrating the efficacy and stability of the suggested method in this study.

**Figure 7:**
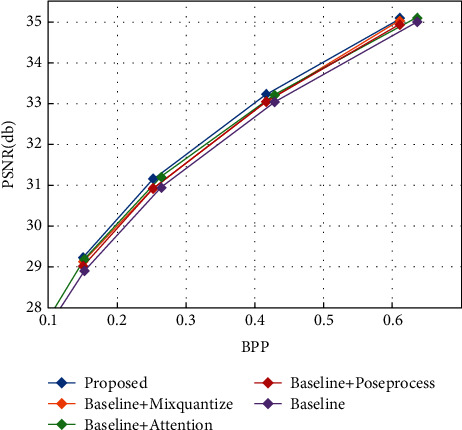
Effectiveness of each module in the proposed method.

We have also conducted ablation studies to validate the effectiveness of each module. As seen in Figure 7, we retrained the model that embeds the hybrid domain attention mechanism into the baseline (four sets of models were trained), and it can see that it produced a rise of around 0.2 db over the baseline model. We also retrained the model by changing the quantization in the baseline to our hybrid quantization (four sets of models were trained), and it can be seen to produce a rise of about 0.2 db over the baseline, with more gain at higher bit rates. We retrained the model with our postprocessing module added to the baseline (four sets of models were trained), and it can be seen to produce a rise of nearly 0.2 db over the baseline as well.

There are four aspects that affect the efficiency of image compression. One is how to extract more compact features. One is the reduction in quantization losses. One is the reconstruction ability of the decoder. One is the accuracy of the entropy estimation. In our method, we improved the baseline model in these four dimensions, and from our ablation experiments, we can conclude that the hybrid domain attention mechanism has the greatest impact on performance.

### 4.3. Visual Comparison

**Figure 8:**
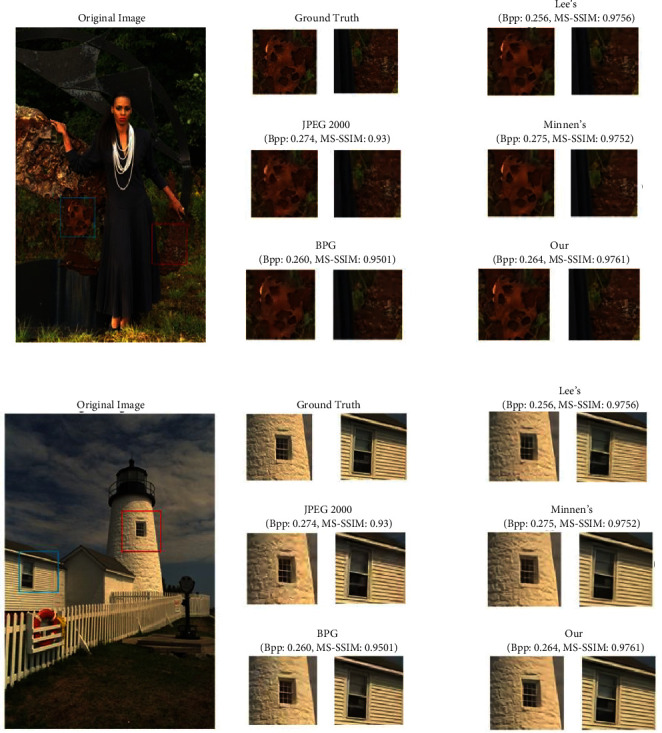
Image comparison with different compression methods under similar compression rates. The pictures are “Kodim18.png” and “Kodim19.png” in the test set Kodak Photo CD dataset [36], respectively.

To make the effectiveness of our framework clearer, we provide some visualization results.  Figure 8 shows the visualization of some images compressed using different compression methods in the Kodak Photo CD dataset [36]. Since neural network-based image compression methods cannot strictly limit the BPP of an image, we can only compare different compression methods at similar BPP of the compressed images. As can be seen from the figures, our method has a higher evaluation index at similar BPP.

In terms of qualitative observation, the image compression methods JPEG 2000 [2] and BPG [3] have obvious block effects and blurring phenomena, and the deep learning-based image compression methods of Lee et al. [11] and Minnen et al. [10] have some loss of edge texture information in the reconstructed images, although they do not have as obvious blurring compression artifacts as the traditional compression methods, while the reconstruction quality of the compression methods in this study is higher. This is because our method embeds a spatial channel mechanism that is accordingly globally adaptive, reinforces important features (e.g., edge texture details), and allocates more bits to important features, and our framework also uses a hybrid quantization mechanism with the addition of an inverse quantization module and a postprocessing module, all of which can guide the network to use fewer bits to obtain higher reconstruction quality. Overall, the approach in this study is also superior in quality of visual comparison.

## 5. Conclusions

We proposed an efficient trainable image compression method and achieve good performance. In particular, we add a hybrid domain attention module that not only improves the transforming capability of the encoder-decoder network but also generates more compact attention masks with hyperprior distribution, which facilitates more accurate probability estimates and thus improves entropy coding efficiency. In addition, our method combines the reuse of intermediate layer information to synthesize the final reconstructed image through a postprocessing enhancement module. We add a quantization adaptive adjustment module to repair quantization losses. This method reduces the total error by optimizing the network parameters through backward propagation. The results of experiments suggest that our method offers a substantial improvement over both traditional compression methods JPEG 2000 [2] and BPG [3], and the learning-based method of Ballé et al. 2017 [6], compared to the advanced learning-based image compression methods of Minnen et al. [10]. This method also has a higher performance index and better visual effect. In the realm of learning-based image compression, extracting compact latent features is especially significant. Attention mechanisms can be still unable to completely eliminate data redundancy in latent features, and better capable attention mechanisms are still being explored. The addition of a postprocessing module on the decoder side and the inverse quantization module in hybrid quantization and the nonlocal block (NLB) [34] in the hybrid domain mechanism all add to the computational complexity, but the performance advantages are significant. We expect that more efficient and lightweight attention mechanisms for extracting latent features can be explored.

## Figures and Tables

**Table 1:** The proposed method's detailed parameter settings. “CONV” signifies convolutional layer. “2↓” signifies downsampling in the stride of 2. “2↑” signifies upsampling in the stride of 2. HDAM represents the hybrid domain attention module.

Encoder	Decoder	Hyperprior encoder	Hyperprior decoder	Context model
CONV:5 × 5 × 128 2↓	DECONV:5 × 5 × 128 2↑	CONV:3 × 3 × 128	DECONV:5 × 5 × 192 2↑	Masked:5 × 5 × 5 × 24

GDN	IGDN	Leaky ReLU	Leaky ReLU	CONV:1 × 1 × 48

CONV:5 × 5 × 128 2↓	DECONV:5 × 5 × 128 2↑	HDAM	HDAM	ReLU

GDN	IGDN	CONV:5 × 5 × 128 2↓	DECONV:5 × 5 × 288 2↑	CONV:1 × 1 × 96

HDAM	HDAM	Leaky ReLU	Leaky ReLU	ReLU

CONV:5 × 5 × 128 2↓	DECONV:5 × 5 × 128 2↑	CONV:5 × 5 × 192 2↓	DECONV:3 × 3 × 384 2↑	CONV:1 × 1 × 1 × 2

GDN	GDN	HDAM		

CONV:5 × 5 × 192 2↓	DECONV:5 × 5 × 3 2↑			

HDAM	HDAM		HDAM	

## Data Availability

The data that support the findings of this study are available from the corresponding author, upon reasonable request.
